# The influence of progressive-chronic and acute sodium bicarbonate supplementation on anaerobic power and specific performance in team sports: a randomized, double-blind, placebo-controlled crossover study

**DOI:** 10.1186/s12986-020-00457-9

**Published:** 2020-05-24

**Authors:** Krzysztof Durkalec-Michalski, Paulina M. Nowaczyk, Jacek Adrian, Joanna Kamińska, Tomasz Podgórski

**Affiliations:** 1 Institute of Human Nutrition and Dietetics, Poznań University of Life Sciences, Wojska Polskiego 31, 60-624 Poznań, Poland; 2 Department of Food and Nutrition, Poznań University of Physical Education, Królowej Jadwigi 27/39, 61-871 Poznań, Poland; 3 Department of Theory and Methodology of Team Sport Games, Poznań University of Physical Education, Królowej Jadwigi 27/39, 61-871 Poznań, Poland; 4 Department od Physiology and Biochemistry, Poznań University of Physical Education, Królowej Jadwigi 27/39, 61-871 Poznań, Poland

**Keywords:** Ergogenic support, Buffering, Acid-base balance, Alkalizing agent, Exercise performance, Sports nutrition

## Abstract

**Background:**

The aims of this study were to verify the effect of progressive-chronic and acute sodium bicarbonate (SB) supplementation on the anaerobic capacity, blood acid-base balance, and discipline-specific performance in team sports disciplines.

**Methods:**

Twenty-four trained male field hockey players completed a randomized, placebo-controlled, crossover trial of either progressive-chronic (increments from 0.05 up to 0.2 g/kg) or an acute one-off dose (0.2 g/kg) supplementation protocol. Before and after treatments, athletes completed an exercise protocol that comprised of a discipline-specific field performance test conducted between two separate Wingate anaerobic tests (WAnTs).

**Results:**

Progressive-chronic SB supplementation improved anaerobic capacity in the first bout of WAnTs, as observed based on an increase in mean power (MP: 575 ± 71 vs. 602 ± 67 W, *p* = 0.005, ~ + 4.7%), peak power (PP: 749 ± 94 vs. 777 ± 96 W, *p* = 0.002, ~ + 3.7%), power carry threshold (P_CT_) at 97%_PP_ (727 ± 91 vs. 753 ± 93 W, *p* = 0.002, ~ + 3.6%) and average power over P_CT_ (739 ± 94 vs. 765 ± 95 W, *p* = 0.001, ~ + 3.5%). Acute SB supplementation had no effect on anaerobic capacity. However, an improvement in time during discipline-specific field performance test was observed after progressive-chronic (919 ± 42 vs. 912 ± 27 s, *p* = 0.05; ~ − 0.8%) and acute (939 ± 26 vs. 914 ± 22 s, *p* = 0.006, ~ 2.7%) SB supplementation. Acute SB supplementation also improved post-exercise parameters of acid-base balance (based on blood pH, bicarbonate concentration and base excess) compared to no supplementation or placebo.

**Conclusions:**

Our study indicates that both chronic and acute SB supplementation positively supports discipline-specific performance among field hockey athletes. Moreover, the chronic protocol supported anaerobic power indices before the inset of exercise-induced fatigue but had no significant impact afterwards. However, only the acute protocol significantly affected the buffering capacity, which can be used to determine athlete’s performance during high-intensity sporting events. This study design therefore highlighted that future studies focusing on sodium bicarbonate supplementation in team sports should concentrate on the efficiency of chronic and acute supplementation in varying time frames.

## Introduction

The use of supplements is common in competitive sports. However, among the numerous preparations, relatively few seem to effectively affect the improvement of physical and exercise capacity [1, 2]. It seems obvious that the decision to enrich the diet with such preparations should aim at solving the problems of limiting the muscle work efficiency during exercise due to exercise-induced changes during training [3]. In our opinion, a serious problem in sport may be also related with muscular acidification induced by intense training/competition effort. This is fundamentally important because muscle acidification affects muscle fatigue related to competition of hydrogen (H^+^) with calcium (Ca^2+^) ions for the troponin binding site, phosphocreatine resynthesis and/or oxidative phosphorylation suppression, inhibition of phosphofructokinase 1 (EC 2.7.1.11) or glycogen phosphorylase (EC 2.4.1.1), a key enzyme of the glycolysis and glycogen degradation, respectively, as well as a decrease in the mitochondrial energy production in muscle cells (due to a reduced mitochondrial matrix-cell cytoplasm proton gradient) [4, 5].

Due to the aforementioned aspects, significant interest in ergogenic support is seen in alkalizing agents like sodium bicarbonate (SB), which may explain its impact on blood alkalosis and increase of extracellular buffer capacity [1, 4, 6]. The supply of alkalizing compounds counteracts these processes, leading to the binding of H^+^ and a greater efflux of H^+^ and lactate from muscle and sustaining muscle contractility during exercise [1, 2, 4, 7]. The benefits of extracellular metabolic alkalosis can also be associated with membrane depolarization, mitochondrial adaptations and acceleration of glycogenolysis, which may enhance exercise performance [8–10].

In previous studies, it was observed that SB supplementation influences the performance, speed, peak and mean muscle power increase, promotes time to reach peak power, total mechanical work, strength endurance, as well as the improvement of sport-specific exercise abilities in speed-strength disciplines and multiple-bouts exercise [1, 4, 11–19]. However, the above mechanisms of action seem to explain the effectiveness of alkalizing agents observed in the studies in sport disciplines of various types of effort lasting mainly ~ 1–4 min, but the results obtained in longer duration efforts are still inconclusive [1, 2, 4, 11, 20]. What is more, there is a lack of knowledge on the extent to which SB supply would affect changes in muscle anaerobic power and specific exercise performance in team sports disciplines where the exercise time is longer, but consists of a relatively large number of intermittent high-intensity periods. In team sports like field hockey, a crucial element is the ability to maintain effective generation and maintenance of power and speed in order to quickly undertake offensive and defensive actions. Muscle work intensity in a hockey match reaches almost maximal efforts followed by short recovery periods. For these reasons the repeated sprint ability for incomplete rest is an important component of fitness for field hockey players. Moreover, it is vital that despite muscular acidification caused by the game (resulting from previous intense actions), players should be able to carry out further efforts effectively [21].

The current state of knowledge does not indicate that SB supplementation is associated with health risks in healthy people [22]. However, in certain cases, excessive intake, especially significantly exceeding recommended doses and/or long-term use, may raise the risk of hypernatremia, electrolyte shifts and systemic pH changes (metabolic alkalosis) [23], and can induce arrhythmias in patients with potassium deficiency [24], tetany in patients with renal failure or/and hypocalcemia [25], systemic alkalosis, and/or central nervous system acidosis [26]. For these reasons, treatment with SB in some clinical cases has to take into account individual health status, water and electrolyte balance and blood bicarbonate concentration. Furthermore, it should be also noted that the major limitation to the most frequently recommended SB doses is its gastrointestinal (GI) side effects, i.e., nausea, diarrhea, bloating, and thirst [22, 27]. Thus, individualized SB supplementation protocols seem to prevent adverse effects, which is important because athletes with whom GI side effects occur may not experience the expected benefits of supplementation [28]. In this respect, the progressive-chronic protocol is safe, but it may be less effective than the most commonly proposed acute protocol [12, 13]. Bearing in mind the lack of knowledge in the above-mentioned areas, as well as the meaningfulness of the practical evaluation of the ergogenic support with SB in team sports athletes, we aimed at assessing the influence of progressive-chronic and acute SB supplementation on the anaerobic power, hockey-specific exercise performance and blood acid-base balance marker concentrations. We hypothesized that both SB supplementation protocols would improve anaerobic power indices; however, the duration of hockey-specific performance test may determine and impair the ergogenic effect of SB treatment in team sports.

## Methods

### Participants

**Fig. 1:**
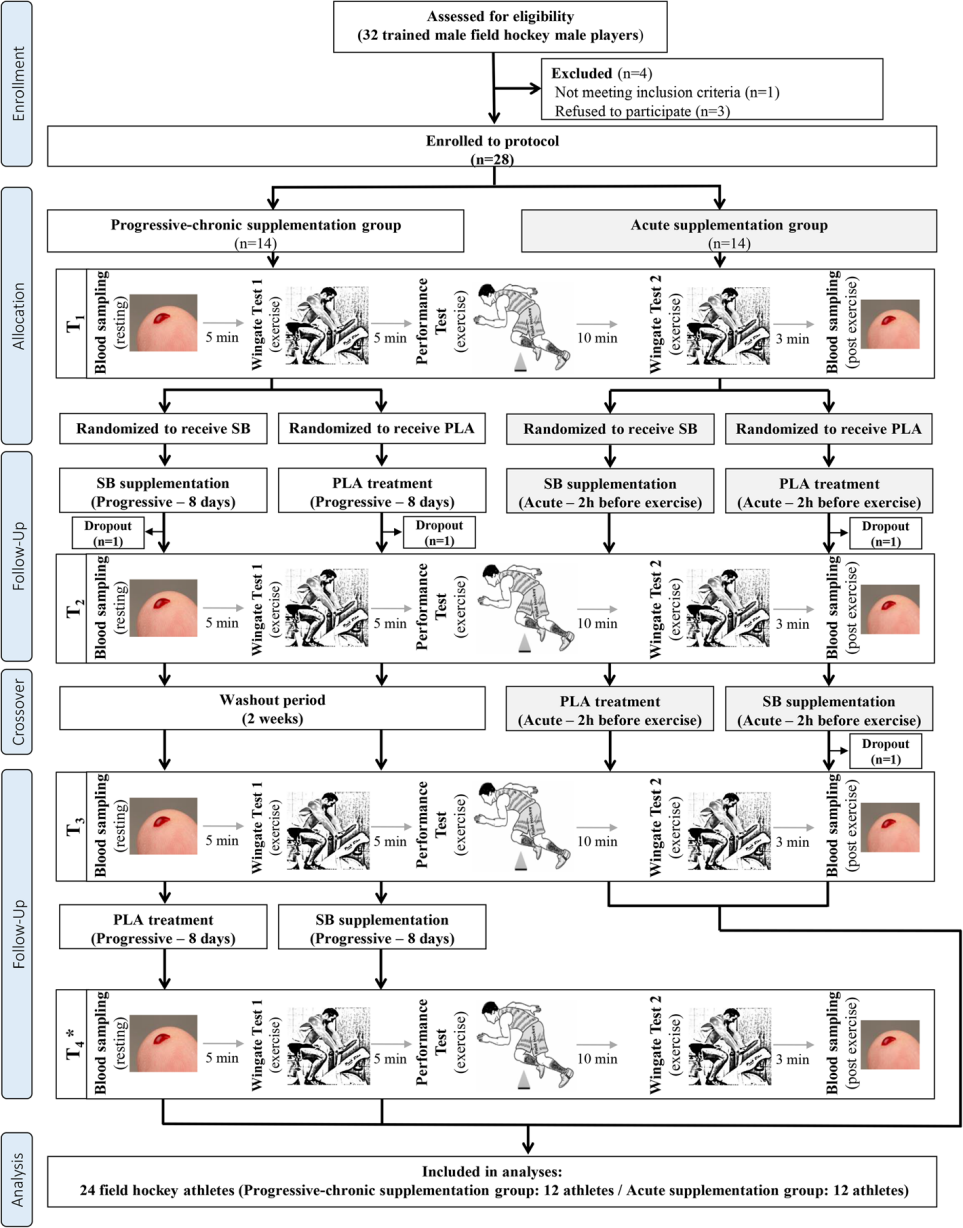
Flow diagram of the study design. Abbreviations: T_1_ − 1st series of test procedures, T_2_ − 2nd series of test procedures, T_3_ − 3rd series of test procedures, T_4_ − 4th series of test procedures, PLA − placebo, SB − sodium bicarbonate

Twenty-eight male field hockey players were initially enrolled in this study. However, 24 athletes finally completed the entire study protocol and were included in the analyses (Fig. 1, Table 1). The participants were members of the Polish Field Hockey National Team and/or Field Hockey League and participated in national and international competitions. The inclusion criteria were a good condition of health, a valid and up-to-date medical certificate confirming the athlete’s ability to practice sports, at least 4 years of training experience, and participation in a minimum of four workout sessions (field hockey) a week (Table S1). The exclusion criteria were current injury, any health-related contraindication, a declared general feeling of being unwell, and unwillingness to follow the study protocol. The dropout rate was relatively small (~ 14%), and four participants did not finish the study protocol (two athletes in the chronic and the acute group, respectively, Fig. 1). However, the dropout reasons were not connected with the study protocol but were related to minor injuries during the customary training practice, which prevented the athletes from participating in exercise tests, and personal reasons.

**Table 1:** Baseline characteristics and typical structure of training loads in tested athletes during chronic and acute supplementation period

	Field hockey players		
	Chronic-group	Acute-group	*p*-Value
n	12	12	–
Age (years)	24.3 ± 5.6	22.2 ± 2.6	0.26^1^
Body mass (kg)	75.0 ± 8.3	82.2 ± 14.1	0.14^2^
Body height (cm)	176 ± 5	183 ± 8	0.02^2^
Body Mass Index (kg/m^2^)	24.2 ± 2.2	24.4 ± 2.3	0.82^2^
Fat mass (%)	12.6 ± 3.2	12.7 ± 4.2	0.94^2^
Fat-free mass (%)	87.4 ± 3.2	87.3 ± 4.2	0.94^2^
Peak power (W)	733 ± 103	792 ± 153	0.27^2^
Mean power (W)	577 ± 73	627 ± 107	0.20^2^
Time in specific performance test (s)	923 ± 42	939 ± 26	0.19^1^
Training sessions per week (no.)	4.7 ± 0.5	4.6 ± 0.7	0.93^1^
Competitions (no.)	1 ± 0	1 ± 0	–
Training time			
total (hours)	8.2 ± 0.9	7.8 ± 1.1	0.04^1^
per one training session (hours)	1.8 ± 0.0	1.7 ± 0.0	0.02^1^

^1^ Data analyzed by Mann−Whitney U test. ^2^ Data analyzed by t-test for independent variables

The primary recruitment strategy was to contact the players’ coaches who enabled the identification and confirmation of required inclusion criteria declared by the participants (such as training experience and the number of training sessions per week). They also supported the compliance of supplementation with the study design. The studies were conducted from December 2018 to July 2019 at the Poznan University of Physical Education. Research was conducted during the preparatory and competitive season. The preparatory period was combined with the start season of indoor hockey. The training microcycle consisted of physical preparation training (three training sessions) and two specialized training sessions in the indoor hall. The training of physical preparation consisted mainly of endurance training − aerobic and strength training. During the start season, most of the training was carried out on the hockey field, including fitness preparation training (interval and speed training) and strength training in the gym. All athletes declared that they had not introduced any changes in their lifestyles, elements of training, nutrition or supplementation, and that they had not been using any medications and supplements with potential ergogenic effects, other than those supplied by the authors of this study. The study was approved by the local ethical committee (Bioethics Committee at Poznan University of Medical Sciences, Poznan, Poland. Decision no. 1000/18 of 11 October 2018) and written informed consent was obtained from all participants before the study began. All procedures were conducted in accordance with the ethical standards of the 1975 Helsinki Declaration. The study complies with the CONSORT Statement for randomized trials as shown in Fig. 1 and Additional file 2 (Table S2. CONSORT checklist).

### Experimental protocol

The influence of supplementation was evaluated in a randomized, crossover, placebo-controlled double-blind trial (Fig. 1). Regarding the double blinding, neither the researchers nor the participants knew whether SB or the placebo (PLA) was administered. Only the head of the department had access to the randomization information, which was only revealed after the cessation of the protocol. The studies consisted of two experimental trials: progressive-chronic and acute SB supplementation protocols. In both aforementioned supplementation protocols the 14-days wash-out period was implemented. The field hockey players were well familiarized with the testing procedures, protocols and equipment used before beginning the study. Anthropometric measures were obtained on the preliminary visit. After qualifying for the study, athletes were subjected to a randomization procedure (in a stratified design with field hockey-specific performance test results being a prognostic variable) and assigned either to the group receiving first a sodium bicarbonate or placebo preparation. The athletes were first enrolled by the authors and then randomly assigned to the supplementation groups with specific codes by an impartial biostatistician. The primary outcomes in our study were changes in mean and peak power capacity and exercise time during field hockey-specific performance test.

### Supplementation

The participants were provided with individually adjusted supplementation schedules. The dose of supplemented sodium bicarbonate was 0.2 g/kg of body mass. In the progressive-chronic supplemented group, the supplementation period lasted 8 days and the dose was increased progressively at 25% every 2 days (from 0.05 g/kg in days 1 and 2 to 0.2 g/kg in days 7 and 8). Furthermore, in this group, the daily dose was also split and ingested 4 times in training days (25% after breakfast, 25% two hours before training, 25% after training and 25% before sleep) or 3 times in non-training day (25% after breakfast, 50% two hours after lunch and 25% before sleep). However, in the acute supplemented group the final dose of 0.2 g/kg was supplemented once for 2 h before using exercise tests. SB was administered in the form of unmarked disk-shaped tablets (Alkala T – (ingredients (per tablet): 1 g sodium bicarbonate and other constituents (content in descending order): lactose, cellulose, potato starch, magnesium stearate, sodium saccharine, gum arabic, maltodextrin, peppermint oil), manufacturer − Sanum Kehbeck GmbH & Co. KG, Germany). The tablets were ingested with at least 500 mL of water. In the placebo (PLA) trial, participants ingested the PLA in a similar tablet form (per tablet instead of sodium bicarbonate 1 g of maltodextrin and 275 mg Na (contained in the added NaCl) were provided.

### Study visits

The field hockey athletes visited the laboratory four times in the chronic (T_1–4_) or three times in the acute (T_1–3_) protocol (Fig. 1). At each visit, body mass and composition were measured, and exercise tests were performed. All the tests were conducted at the Poznan University of Physical Education. The subjects were instructed not to participate in any high-intensity or long-duration training session at least 24 h before testing. The tests were performed in the afternoon hours corresponding to the usual time of athletes’ training units. During all examinations, room temperature remained at 20–21 °C.

### Anthropometric measurements

At the preliminary visit to the laboratory and before exercise tests, anthropometric measurements were taken with the participants in a fasted state during the morning hours. Body mass and height were measured using a professional medical scale with a stadiometer (WPT 60/150 OW, RADWAG®, Radom, Poland). The stadiometer had an accuracy of 0.1 cm and 0.1 kg for height and body mass, respectively. Body fat and fat-free mass were assessed by means of bioelectric impedance, with Bodystat 1500 (Bodystat Inc., Douglas, UK) [29].

### Exercise tests

During each exercise session, all athletes performed two Wingate anaerobic tests (WAnTs) interspersed with a discipline-specific field performance test.

Hockey-specific performance capacity was measured using a specific hockey field test (HST), modified to reflect hockey match activity and of a structure similar to during hockey matches. During the test, the Polar Team^2^ System (POLAR ELECTRO, Kampele, Finland) was used to record the player’s heart rate. The test consisted of running sections based of various distance lengths and uniform recovery breaks:

- 25-yard line and back (45.7 m), and 60 s recovery
- halfway line and back (91.4 m), and 60 s recovery
- 75-yard line and back (137.2 m), and 60 s recovery
- full-length pitch and back (182.9 m), and 60 s recovery
- half-pitch lap (201.2 m), and 60 s recovery
- full-pitch lap (292.6 m), and 60 s recovery
- half-pitch lap (201.2 m), and 60 s recovery
- full-length pitch and back (182.9 m), and 60 s recovery
- 75-yard line and back (137.2 m), and 60 s recovery
- halfway line and back (91.4 m), and 60 s recovery
- 25-yard line and back (45.7 m), and 60 s recovery

The stopwatch was stopped as the crossed the baseline (10 min recovery were subtracted from the total HST time).

Anaerobic power was assessed using the classical WAnT on a cycloergometer (Monark 894E, Varberg, Sweden), following the recommendations for such tests as proposed by Bar-Or [30]. The WAnT was performed twice; the first (WAnT_1) 5 min before and the second (WAnT_2) 10 min after the HST (Fig. 1). The seat height was adjusted to each participant’s satisfaction and toe clips with straps were used to prevent the feet from slipping off the pedals. The primary test was preceded by a 5 min warm-up period of approximately 50 W power. This was followed by two run-up practices of 3 s, during which the actual test load was imposed to enable the participants to become accustomed to the resistance. The test lasted for 30 s. External loading was estimated individually at 7.5% body weight. During the test, the athletes were encouraged to exert maximum effort. The recorded results included the work effort (W_EF_), the mean (MP) and peak (PP) power output, power carry threshold (P_CT_) at 97%_PP_ and average power over the P_CT_ (AP_CT_), which were analyzed using MCE v_5.1 software (“JBA” Zb.Staniak (C), Warsaw, Poland).

### Blood samples analysis

At rest (EXERCISE__PRE_) and 3 min after the last effort test (EXERCISE__POST_) capillary blood was collected from a fingertip of the nondominant hand using a disposable lancet-spike Medlance® Red (HTL-STREFA, Łódź, Poland) with a 1.5 mm blade and 2.0 mm penetration depth. Approximately 65 μl of blood was collected to a heparinized capillary tube where lactate (La), bicarbonate (HCO_3_¯), base excess (BE) concentrations and pH value were determined on blood gas analyzer (ABL90 FLEX, Radiometer, Brønshøj, Denmark).

### Statistical analysis

All variables were checked for normal distribution using the Shapiro−Wilk test. Differences in baseline characteristics between chronic-group and acute-group were tested by t-test for independent variables (for normally distributed data; effect size expressed as Cohen’s *d*; 0.20 – small effect, 0.50 – medium effect and 0.80 – large effect) or Mann−Whitney U test (variables not normally distributed; effect size expressed as Glass’s rank-biserial correlation coefficient (*r*_*g*_); interpretation according to correlation coefficient). In the case of the chronic supplementation strategy, “pre-post” supplementation differences in results of WAnT_1 and WAnT_2, or in results of HST and parameters of acid-base balance were tested by two-way (“pre-post” x sequence of SB and PLA administration) ANOVA with repeated measurements (normal distribution) or Wilcoxon signed-rank test (not normal distribution). The corresponding results of acute supplementation strategy were tested by one-way ANOVA with repeated measurements (normal distribution) or Friedman’s ANOVA and subsequent two-group comparisons by Wilcoxon signed-rank test (not normal distribution). Effect sizes were calculated as partial eta-squared (${\eta}_p^2$; 0.01 – small effect, 0.06 – medium effect and 0.14 – large effect) or Kendall’s concordance coefficient *W* (0 – no agreement and 1 – complete agreement), respectively. G*Power software (version 3.1.9.4, Universität Düsseldorf, Germany) was used to calculate sample size required to obtain a power of approximately 80% (α = 0.05) and a large effect size partial eta-squared 0.14 in analysis of variance (ANOVA) with repeated measurements within factors. Analysis indicated that a sample size of 10 and 12 would be suitable for detecting a difference between four and three measurements, respectively. Statistical significance was set at *p* < 0.05, and data were analyzed using the STATISTICA-13.3 software program (StatSoft Inc., USA).

## Results

### Chronic supplementation strategy

Chronic SB supplementation substantially improved performance in WAnT_1, but not WAnT_2 (Table 2). W_EF,_ MP, PP, P_CT_ at 97%_PP_ and AP_CT_ were also significantly higher at SB_-POST_ compared to SB_-PRE_ in WAnT_1. Furthermore, chronic PLA supplementation resulted only in significantly higher W_EF_ in WAnT_1.

**Table 2:** Results of Wingate anaerobic test before (WAnT_1) and after (WAnT_2) hockey-specific performance test – chronic supplementation strategy

		SB_-PRE_	SB_-POST_	*p*-Value *Z*^1^ or *F*^2^ *r*_*c*_^*1*^ or ${\eta}_p^2$^2^	PLA_-PRE_	PLA_-POST_	*p*-Value *Z*^1^ or *F*^2^ *r*_*c*_^*1*^ or ${\eta}_p^2$^2^
Work effort (W)	WAnT _1	232 ± 19	243 ± 16	0.005^1^	234 ± 14	241 ± 14	0.031^2^
				2.82^1^			6.257^2^
				0.82^1^			0.385^2^
	WAnT _2	225 ± 23	228 ± 19	0.365^2^	227 ± 14	238 ± 17	0.104^2^
				0.898^2^			3.205^2^
				0.082^2^			0.243^2^
Mean power (W)	WAnT _1	575 ± 71	602 ± 67	0.005^2^	585 ± 73	602 ± 74	0.264^2^
				12.50^2^			1.397^2^
				0.556^2^			0.123^2^
	WAnT _2	560 ± 90	567 ± 77	0.382^2^	569 ± 72	595 ± 74	0.393^2^
				0.837^2^			0.796^2^
				0.077^2^			0.074^2^
Peak power (W)	WAnT _1	749 ± 94	777 ± 96	0.002^2^	753 ± 105	784 ± 104	0.254^2^
				17.306^2^			1.464^2^
				0.634^2^			0.128^2^
	WAnT _2	714 ± 109	738 ± 117	0.182^1^	723 ± 89	762 ± 105	0.222^2^
				1.334^1^			1.699^2^
				0.38^1^			0.145^2^
Time to PP (s)	WAnT _1	6.10 ± 1.06	5.84 ± 0.94	0.246^2^	6.28 ± 0.87	5.98 ± 1.12	0.942^2^
				1.519^2^			0.006^2^
				0.132^2^			0.001^2^
	WAnT _2	5.77 ± 0.89	6.19 ± 1.16	0.766^2^	6.84 ± 2.46	5.88 ± 0.96	0.232^2^
				0.093^2^			1.618^2^
				0.009^2^			0.139^2^
Power carry threshold at 97%_PP_ (W)	WAnT _1	727 ± 91	753 ± 93	0.002^2^	731 ± 102	761 ± 101	0.244^2^
				17.191^2^			1.532^2^
				0.632^2^			0.133^2^
	WAnT _2	693 ± 106	716 ± 114	0.182^1^	701 ± 86	740 ± 102	0.228^2^
				1.334^1^			1.650^2^
				0.38^1^			0.142^2^
Average power over the P_CT_ (W)	WAnT _1	739 ± 94	765 ± 95	0.001^2^	742 ± 104	774 ± 102	0.230^2^
				21.444^2^			1.632^2^
				0.682^2^			0.140^2^
	WAnT _2	704 ± 108	728 ± 116	0.182^1^	713 ± 87	752 ± 102	0.197^2^
				1.334^1^			1.910^2^
				0.380^1^			0.160^2^
HR_max_ (bpm)	WAnT _1	175 ± 8	172 ± 9	0.160^2^	173 ± 9	171 ± 9	0.158^1^
				2.300^2^			1.412^1^
				0.187^2^			0.408^1^
	WAnT _2	179 ± 6	177 ± 9	0.683^2^	177 ± 6	179 ± 8	0.182^1^
				0.177^2^			1.334^1^
				0.017^2^			0.385^1^

^1^Data analyzed by Wilcoxon signed-rank test; effect size expressed as rank correlation coefficient (*r*_*c*_). ^2^Data analyzed by two-way ANOVA with repeated measurements; effect size expressed as partial eta-squared (${\eta}_p^2$). Abbreviations: HR_max_ – maximal heart rate, P_CT_ – power carry threshold at 97%_PP_, PLA_-PRE_ – before placebo treatment, PLA_-POST_ – after placebo treatment, PP – peak power, SB_-PRE_ – before sodium bicarbonate supplementation, SB_-POST_ – after sodium bicarbonate supplementation, WAnT_1 – the Wingate anaerobic test performed before hockey-specific performance test, WAnT_2 – the Wingate anaerobic test performed after hockey-specific performance test

**Fig. 2:**
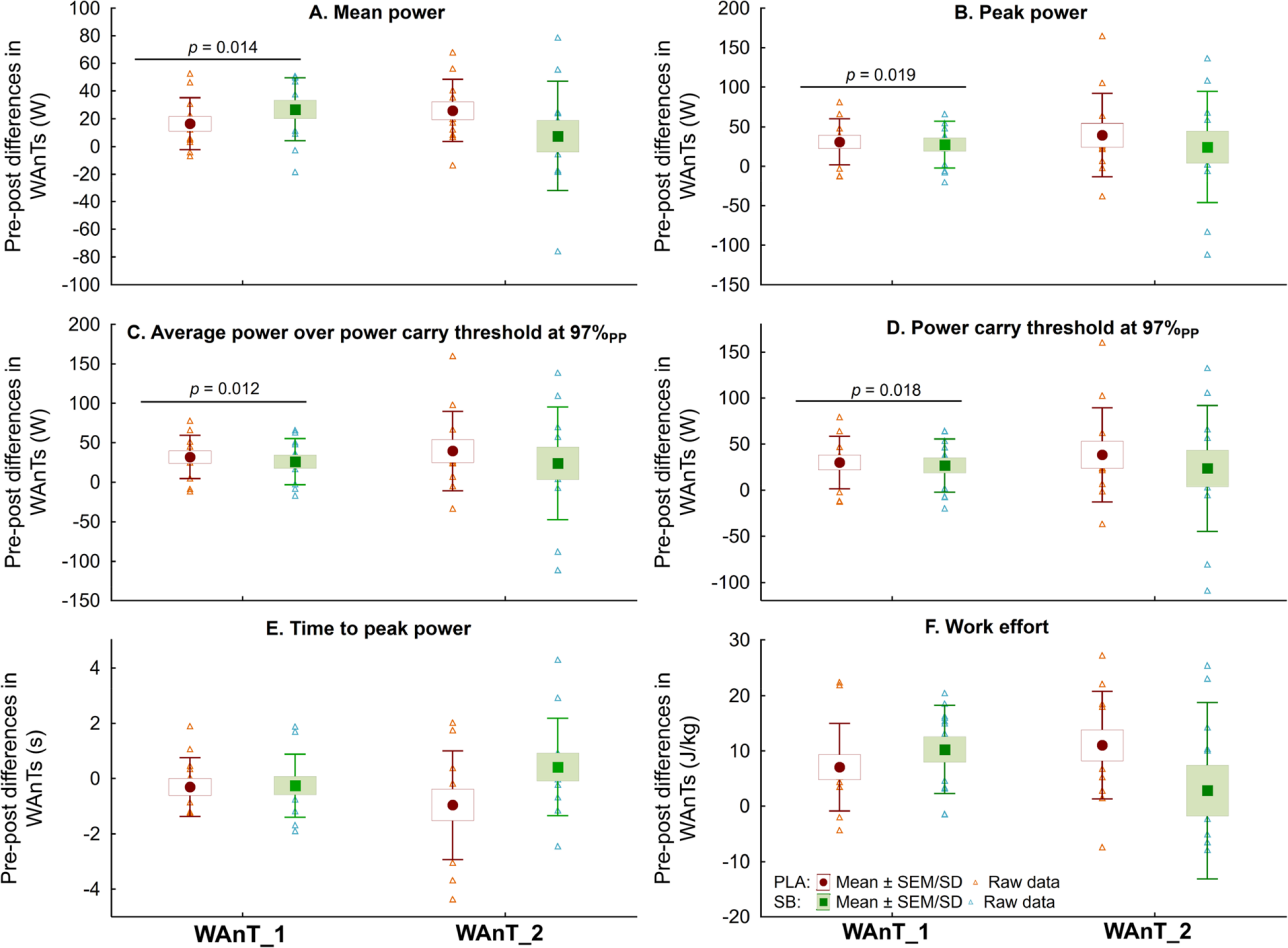
Comparisons of “pre-post” absolute differences in WAnTs (“After” – “Before” treatment) between SB and PLA – chronic supplementation strategy. Abbreviations: PLA – placebo treatment, PP – peak power, SB – sodium bicarbonate supplementation, WAnT_1 – the Wingate anaerobic test performed before hockey-specific performance test, WAnT_2 – the Wingate anaerobic test performed after hockey-specific performance test

Analysis of absolute change of evaluated markers (Δ _PRE-POST_) indicated an increase in MP in WAnT_1, which was higher after SB, compared to PLA supplementation (Fig. 2a). However, increases in PP (Fig. 2b), AP_CT_ (Fig. 2c) and P_CT_ (Fig. 2d) were substantially higher after PLA compared to SB.

Moreover, SB supplementation resulted in significant improvement in specific-performance (reduction in time of HST), while no changes were observed in HR (Table 3). In addition, PLA supplementation had no effect on HST performance or HR.

**Table 3:** Results in hockey-specific performance test – chronic supplementation strategy

	SB_-PRE_	SB_-POST_	*p*-Value *Z*^1^ or *F*^2^ *r*_*c*_^1^ or ${\eta}_p^2$^2^	PLA_-PRE_	PLA_-POST_	*p*-Value *Z*^1^ or *F*^2^ *r*_*c*_^1^ or ${\eta}_p^2$^2^	Δ SB _PRE-POST_	Δ PLA _PRE-POST_	*p*-Value *Z*^1^ or *F*^2^ r_c_^1^ or ${\eta}_p^2$^2^
Time (s)	919 ± 42	912 ± 27	0.050^1^	919 ± 33	920 ± 31	0.088^2^	−6.5 ± 53.1	0.5 ± 32.6	0.638^1^
			1.956^1^			3.576^2^			0.471^1^
			0.590^1^			0.263^2^			0.136^1^
HR_max_ (bpm)	179 ± 5	179 ± 7	0.756^1^	179 ± 9	179 ± 7	0.251^2^	−0.3 ± 3.9	−0.7 ± 9.4	0.424^1^
			0.311^1^			1.485^2^			0.800^1^
			0.094^1^			0.129^2^			0.241^1^
HR_average_ (bpm)	160 ± 6	159 ± 7	0.266^1^	159 ± 7	160 ± 7	0.644^2^	−1.2 ± 4.7	1.0 ± 4.7	0.963^2^
			1.111^1^			0.227^2^			0.002^2^
			0.335^1^			0.022^2^			0.000^2^

^1^Data analyzed by Wilcoxon signed-rank test; effect size expressed as rank correlation coefficient (r_c_). ^2^Data analyzed by two-way ANOVA with repeated measurements; effect size expressed as partial eta-squared (${\eta}_p^2$). Abbreviations: HR_average_ – average heart rate, HR_max_ – maximal heart rate, PLA_-PRE_ – before placebo treatment, PLA_-POST_ – after placebo treatment, SB_-PRE_ – before sodium bicarbonate supplementation, SB_-POST_ – after sodium bicarbonate supplementation

### Acute supplementation strategy

Acute supplementation strategy had no effect on WAnTs (Table 4), but substantially increased performance in HST (Table 5). Additionally, average HR was decreased after SB and PLA compared to baseline.

**Table 4:** Results of Wingate anaerobic test before (WAnT_1) and after (WAnT_2) hockey-specific performance test – acute supplementation strategy

		BASE	SB	PLA	*p-*Value *Χ*^2 2^ or *F*^1^ Kendall’s *W*^2^ or ${\eta}_p^2$^1^
Work effort (W)	WAnT _1	229 ± 14	229 ± 14	226 ± 12	0.397^1^
					0.823^1^
					0.070^1^
	WAnT _2	215 ± 21	225 ± 18	217 ± 20	0.106^1^
					2.492^1^
					0.185^1^
Mean power (W)	WAnT _1	627 ± 107	636 ± 105	626 ± 104	0.383^1^
					0.877^1^
					0.074^1^
	WAnT _2	670 ± 71	700 ± 48	675 ± 66	0.146^1^
					2.102^1^
					0.160^1^
Peak power (W)	WAnT _1	792 ± 153	813 ± 146	783 ± 154	0.066^1^
					3.081^1^
					0.219^1^
	WAnT _2	849 ± 72	873 ± 75	857 ± 81	0.516^1^
					0.682^1^
					0.058^1^
Time to PP (s)	WAnT _1	6.19 ± 1.67	6.40 ± 1.78	6.30 ± 1.60	0.760^1^
					0.278^1^
					0.025^1^
	WAnT _2	8.00 ± 4.46	6.45 ± 2.40	7.04 ± 1.53	0.558^2^
					1.167^2^
					0.049^2^
Power carry threshold at 97%_PP_ (W)	WAnT _1	769 ± 149	788 ± 142	760 ± 150	0.065^1^
					3.104^1^
					0.220^1^
	WAnT _2	823 ± 70	846 ± 73	831 ± 79	0.516^1^
					0.681^1^
					0.058^1^
Average power over the P_CT_ (W)	WAnT _1	782 ± 151	801 ± 144	772 ± 152	0.063^1^
					3.137^1^
					0.222^1^
	WAnT _2	838 ± 71	860 ± 74	845 ± 80	0.562^1^
					0.592^1^
					0.051^1^
HR_max_ (bpm)	WAnT _1	170 ± 12	166 ± 10	168 ± 11	0.152^2^
					3.762^2^
					0.171^2^
	WAnT _2	175 ± 10	174 ± 7	174 ± 10	0.947^1^
					0.054^1^
					0.006^1^

^1^Data analyzed by one-way ANOVA with repeated measurements; effect size expressed as partial eta-squared (${\eta}_p^2$). ^2^Data analyzed by Friedman’s ANOVA and subsequent two-group comparisons by Wilcoxon signed-rank test; effect size expressed as Kendall’s Concordance Coefficient *W.* Abbreviations: BASE – baseline (no supplementation), HR_max_ – maximal heart rate, P_CT_ – power carry threshold at 97%_PP,_ PLA – after placebo treatment, PP – peak power, SB – after sodium bicarbonate supplementation, WAnT_1 – the Wingate anaerobic test performed before hockey-specific performance test, WAnT_2 – the Wingate anaerobic test performed after hockey-specific performance test

**Table 5:** Results in hockey-specific performance test – acute supplementation strategy

	BASE	SB	PLA	*p-*Value *F* ${\eta}_p^2$
Time (s)	939 ± 26^b^	914 ± 22^a^	919 ± 20^a^	0.006
				6.480
				0.371
HR_max_ (bpm)	179 ± 7	178 ± 7	178 ± 8	0.260
				1.453
				0.139
HR_average_ (bpm)	163 ± 11^b^	157 ± 7^a^	160 ± 12^a^	0.004
				7.423
				0.452

^ab^ – different letter inscriptions refer to statistical differences between BASE, PLA and SB. Abbreviations: BASE – baseline (no supplementation), HR_average_ – average heart rate, HR_max_ – maximal heart rate, PLA – after placebo treatment, SB – after sodium bicarbonate supplementation

### Blood acid-base balance marker analysis

Chronic SB supplementation had no vital effect on blood acid-base balance (Table 6). On the contrary, acute supplementation strategy significantly affected post-exercise acid-base balance tests (Table 7) compared to no supplementation (BASE) or PLA.

**Table 6:** Blood acid-base balance markers – chronic supplementation strategy

		SB_-PRE_	SB_-POST_	*p-*Value *T*^1^ or *Z*^2^ *d*^1^ or *r*_*c*_^2^	PLA_-PRE_	PLA_-POST_	*p-*Value *T*^1^ or *Z*^2^ *d*^1^ or *r*_*c*_^2^	SB_-POST_ vs. PLA_-POST_ *p-*Value *T*^3^ or *U*^4^ *d*^3^ or *r*_*g*_^4^
pH	EXERCISE__PRE_	7.41 ± 0.02	7.43 ± 0.02	0.101^1^	7.42 ± 0.02	7.43 ± 0.03	0.183^1^	0.768^3^
				−1.792^1^			−1.421^1^	0.298^3^
				−0.652^1^			− 0.432^1^	0.122^3^
	EXERCISE__POST_	7.18 ± 0.05	7.20 ± 0.05	0.368^1^	7.18 ± 0.05	7.18 ± 0.04	0.921^1^	0.314^3^
				−0.939^1^			−0.102^1^	1.031^3^
				−0.390^1^			−0.032^1^	0.421^3^
HCO_3_*¯* (mmol/L)	EXERCISE__PRE_	26.1 ± 0.8	27.2 ± 2.1	0.068^1^	26.3 ± 1.2	26.5 ± 1.6	0.906^2^	0.166^4^
				−2.021^1^			0.118^2^	47.500^4^
				−0.719^1^			0.034^2^	0.340^4^
	EXERCISE__POST_	13.1 ± 1.6	13.6 ± 1.5	0.502^1^	13.4 ± 1.6	13.2 ± 1.3	0.670^1^	0.490^3^
				−0.695^1^			0.438^1^	0.703^3^
				−0.306^1^			0.162^1^	0.287^3^
La (mmol/L)	EXERCISE__PRE_	1.6 ± 0.5	1.4 ± 0.7	0.147^2^	1.4 ± 0.4	1.4 ± 0.7	0.666^2^	0.885^4^
				1.451^2^			0.431^2^	69.000^4^
				0.815^2^			0.125^2^	0.042^4^
	EXERCISE__POST_	17.7 ± 2.5	17.5 ± 2.8	0.799^2^	17.0 ± 2.5	17.0 ± 1.8	0.986^1^	0.707^4^
				0.255^2^			−0.018^1^	65.000^4^
				0.074^2^			− 0.008^1^	−0.097^4^
BE (mmol/L)	EXERCISE__PRE_	2.2 ± 1.0	3.6 ± 2.6	0.073^1^	2.4 ± 1.5	2.6 ± 2.0	1.000^2^	0.166^4^
				−1.981^1^			0.000^2^	47.500^4^
				−0.683^1^			0.000^2^	0.340^4^
	EXERCISE__POST_	−16.9 ± 2.7	−16.0 ± 2.6	0.453^1^	−16.2 ± 3.0	−16.7 ± 2.5	0.658^1^	0.495^3^
				−0.778^1^			0.455^1^	0.694^3^
				−0.341^1^			0.158^1^	0.283^3^

^1^Data analyzed by t-test for dependent variables; effect size expressed as Cohen’s *d*. ^2^Data analyzed by Wilcoxon signed-rank test; effect size expressed as rank correlation coefficient (*r*_*c*_). ^3^Data analyzed by t-test for independent variables; effect size expressed as Cohen’s *d*. ^4^Data analyzed by Mann−Whitney U test; effect size expressed as Glass’s rank-biserial correlation coefficient (*r*_*g*_). Abbreviations: BE – base excess, EXERCISE__PRE_ – value before exercise tests, EXERCISE__POST_ – value 3-min after the second Wingate anaerobic test, HCO_3_¯– bicarbonate, La – blood lactate, PLA_-PRE_ – before placebo treatment, PLA_-POST_ – after placebo treatment, SB_-PRE_ – before sodium bicarbonate supplementation, SB_-POST_ – after sodium bicarbonate supplementation

**Table 7:** Blood acid-base balance markers – acute supplementation strategy

		BASE	SB	PLA	*p-*Value *F*^1^ or *Χ*^*2* 2^ ${\eta}_p^2$^1^ or Kendall’s *W*^2^
pH	EXERCISE__PRE_	7.41 ± 0.02	7.41 ± 0.03	7.41 ± 0.02	0.593^1^
					1.000^1^
					0.046^1^
	EXERCISE__POST_	7.18 ± 0.04^a^	7.24 ± 0.05^b^	7.18 ± 0.04^a^	0.000^2^
					15.500^2^
					0.646^2^
HCO_3_*¯* (mmol/L)	EXERCISE__PRE_	25.9 ± 1.0	26.1 ± 1.2	25.6 ± 0.8	0.558^2^
					1.167^2^
					0.049^2^
	EXERCISE__POST_	13.3 ± 1.5^a^	15.4 ± 1.9^b^	13.2 ± 1.6^a^	0.000^2^
					18.167^2^
					0.757^2^
La (mmol/L)	EXERCISE__PRE_	1.3 ± 0.33	1.3 ± 0.5	1.2 ± 0.5	0.850^2^
					0.326^2^
					0.014^2^
	EXERCISE__POST_	16.4 ± 2.5^b^	17.2 ± 2.2^b^	15.2 ± 3.0^a^	0.009^2^
					9.456^2^
					0.394^2^
BE (mmol/L)	EXERCISE__PRE_	2.4 ± 1.1	2.1 ± 1.4	2.2 ± 1.1	0.920^2^
					0.167^2^
					0.007^2^
	EXERCISE__POST_	−16.1 ± 2.7^a^	−13.0 ± 3.3^b^	−16.4 ± 2.9^a^	0.000^2^
					18.000^2^
					0.750^2^

^1^Data analyzed by one-way ANOVA with repeated measurements; effect size expressed as partial eta-squared (${\eta}_p^2$); ^2^Data analyzed by Friedman’s ANOVA and subsequent two-group comparisons by Wilcoxon signed-rank test; effect size expressed as Kendall’s Concordance Coefficient *W*; ^ab^ – different letter inscriptions refer to statistical differences between BASE, PLA and SB. Abbreviations: BASE – baseline (no supplementation), BE – base excess EXERCISE__PRE_ – value before exercise tests, EXERCISE__POST_ – value 3 min after the second Wingate anaerobic test, HCO_3_*¯*- bicarbonate, La – blood lactate, PLA – after placebo supplementation, SB – after sodium bicarbonate supplementation

Blood pH was higher after SB compared to BASE and PLA. Simultaneously, HCO_3_¯ concentration and BE were significantly higher after SB compared to BASE and PLA. However, there were no differences in La concentration between BASE and SB. Moreover, La concentration was substantially lower after PLA compared to BASE and SB (Table 7).

## Discussion

In this study in trained field hockey players, an acute or progressive-chronic SB supplementation strategy versus placebo was implemented to investigate the effectiveness of the two different treatment strategies in improving an anaerobic capacity at rest and after discipline-specific exercises, the discipline-specific performance, and the acid-base balance. Thus, to our knowledge, this is the first study to examine the issue of SB supplementation in team-sports players with such a broad approach.

Solely the chronic SB supplementation had the potential to improve the anaerobic capacity (in the Wingate anaerobic test) in studied athletes, and the effect was exclusive for the tests performed before (WAnT_1) but not after HST exercise. Simultaneously, it is worth underlining that both supplementation strategies were effective in improving specific hockey performance in HST. However, regrading acute supplementation strategy, PLA was surprisingly equally effective as SB.

Acute and chronic SB supplementation strategies have been studied with respect to anaerobic capacity in earlier studies [4, 11, 13–19, 31, 32]. Nevertheless, the variety of methodologies applied in the studies is extremely high, starting from studied groups (healthy non-athletes vs. athletes of various sport disciplines), duration of supplementation periods (acute vs. various chronic supplementation protocols), doses of SB supplementation or using SB in conjunction with other supplements, or tests applied for evaluating anaerobic capacity. Regarding the latter, the WAnT [30] is one of the most commonly utilized tests to evaluate high-intensity exercise capacity and power. A recent meta-analysis by Lopes-Silva and colleagues [32] aimed at evaluating the effectiveness of the acute and chronic SB supplementation on anaerobic performance in individual and multiple bouts of WAnTs. The meta-analysis included nine studies, of which six investigated acute, and only three studies chronic supplementation strategies. The number of WAnT bouts in the analyzed studies ranged from 1 to 4 (with 3–15 min of recovery between bouts). It was revealed that acute SB ingestion did not improve PP or MP in WAnT regardless of the number of bouts performed. On the contrary, the chronic SB supplementation strategies were effective in improving PP and MP in all bouts of WAnT performed. In general, the findings by Lopes-Silva et al. [32] are in line with the results of our study – where we registered an increase in PP and MP after chronic SB supplementation in the first bout of WAnT. However, Lopes-Silva and colleagues [32] noticed that within the meta-analysis, the effect size of performance improvements, especially with regard to PP, after chronic SB supplementation increased throughout successive bouts of WAnTs. It should be noticed that the statistical analysis for PP and chronic supplementation strategies included only two studies. The authors of this meta-analysis suggested that regarding the mechanisms responsible for the ergogenic effect of SB, it is more expected for chronic SB supplementation to improve PP in repeated bouts of WAnT (the second bout in the raw and the following ones) than in the first/one single bout of WAnT, while improvement in MP could be expected throughout single and multiple bouts of WAnT. In our study, the participants performed two bouts of WAnT during each testing day. However, according to the experimental protocol and objectives of our study, the participants were subjected to performing HST in between the two bouts of WAnTs (WAnT_1 and WAnT_2). In these experimental conditions, contrary to the conclusions by Lopes-Silva et al. [32] and simultaneously not supporting the hypothesis of our study, the progressive-chronic SB supplementation was no longer effective in improving PP or MP, or any other parameter measured during the test in the second bout of WAnT (WAnT_2). Although chronic SB supplementation did not result in substantial changes in the markers of the blood acid-base balance before and after exercises, a clear base deficit (as measured by BE) was seen after exercise tests comparing to resting value. It seems reasonable that the lack of sustained changes in the blood acid-base balance may be the cause of a lack of ergogenic effect of chronic SB ingestion in anaerobic capacity after exercise (in WAnT_2).

Only one of the studies included in the discussed meta-analysis [32] encompassed team sport athletes − rugby players − alongside combat sports athletes, as participants in a studied group [33]. Although the effectiveness of SB supplementation on anaerobic capacity or discipline-specific performance has been studied in various groups of athletes, data on team sports, and in particular on field hockey players is scarce, and limited to female athletes [34]. In general, team sports (e.g., soccer, basketball, handball, field hockey, futsal, volleyball) are multidirectional sports, requiring different volume and proportion of sagittal plane sprinting and high-intensity running, lateral shuffling or cutting, and some are heavily reliant on jumping [35]. They are characterized by the need to perform bouts of high-intensity exercise interspersed with short periods of recovery, accompanied by correct decision-making and technical execution of sports-specific skills [34]. Thus, the discipline-specific performance in team sport is often evaluated based on various running-based tests of multiple sprints [36]. Macutkiewicz et al. [34] found that a single dose of 0.3 g/kg BW of SB did not improve sprint or sport-specific skill performance, though it resulted in a lower rate of perceived exertion in female field hockey players. The latter may have performance implications in a competitive match situation. Similarly, Cameron et al. [37] noted that 0.3 g/kg BW of SB did not improve performance in rugby-specific repeated-sprint test in elite male rugby players. Cholewa et al. [36] observed no improvement in performance in the Yo-Yo Intermittent Recovery Test Level 2 after the single ingestion of 0.3 g/kg BW of SB in male soccer players. In contrast, Ducker et al. 2013 [38] found that acute SB supplementation (0.3 g/kg BW) resulted in better Repeated-Sprint Test performance compared to chronic beta-alanine (28 days), acute SB in conjunction with chronic beta alanine, or placebo supplementation in male team-sport athletes (Australian football, field hockey, soccer). Bishop and Claudius [39] in female team-sport athletes noted that a single dose of 0.2 g/kg BW of SB may also have an ergogenic effect during the second-half performance of the Intermittent-Sprint Test (IST; a test designed to replicate the average sprint profile of a typical team-sport game). Furthermore, it was based on completing significantly more work in 7 of 18 s-half sprint ISTs. Nevertheless, the total work completed and mean peak power achieved were not significantly different between SB and placebo in either half of the IST. In the latest work from our lab [11] we also observed that chronic SB supplementation can lead to the maintenance of high anaerobic power mainly in the midsection of the 30 s Wingate test; however, the specific-performance (in wrestling) may be gender-specific and seems to be more effective in male than female athletes. Furthermore, taken together, the results of most of the studies investigating acute SB supplementation on discipline-specific performance in team sports remain partially in contrast with the results of our study. In our study SB was more effective than no supplementation in improving time in the hockey-specific performance test. However, PLA was as equally effective as SB. It could be worth considering the fact that we used maltodextrin (with NaCl) as a PLA, as did Macutkiewicz et al. [34]; while other authors used glucose [38], cornflower [36], or NaCl [37, 39]. The use of particular substances as PLA in the placebo-controlled trials may have some implications in the results of the studies. Performance of intermittent sports is dependent upon a combination of anaerobic and aerobic energy systems, both of which rely on carbohydrates as an important fuel source [40]. In our study, the presence of carbohydrates in the PLA was connected with the ingestion of 15 ± 2 g of additional carbohydrates (depending on body mass from 13 to 19 g) in comparison to their intake during SB supplementation (and no supplementation), and might have resulted in a slower depletion of muscle glycogen storage, a lower perception of temporary and permanent fatigue, and eventually led to an increased performance [40]. The results obtained in our study suggest that it might be reasonable to investigate the combined effect of acute SB and carbohydrate ingestion on performance in the intermittent sports.

To our knowledge there are no earlier studies on chronic SB supplementation in team sports. In the study by Chycki et al. [41] in elite soccer players, SB was supplemented in combination with potassium dicarbonate and minerals – calcium phosphate, calcium citrate, potassium citrate and magnesium citrate. It was then found that 9 days of supplementation was effective in improving anaerobic performance as measured by the Running-Based Anaerobic Sprint Test.

Finally, it should also be taken into account that the duration of effort may be a crucial trigger related to SB efficiency. Hadzic et al. [42] indicate duration of exercise as a factor determining the ergogenic effect of SB. The authors performed a systematic review of 35 studies on the effect of acute (31 studies) or chronic (5 studies) SB supplementation on performance in exercises lasting ≤ 4 min (17 studies with acute supplementation and 4 studies with chronic supplementation) or > 4 min (14 studies with acute supplementation and 1 study with chronic supplementation) in athletes of various sport disciplines. With regard to exercises lasting ≤ 4 min, an indisputable enhancement in performance was seen in nine studies with acute supplementation and two studies with chronic supplementation; while divergent effects (e.g. enhancement in one out of a few exercise tasks or enhancement after a combination of SB with other supplements) were seen in three and one studies, respectively. With regard to exercises lasting > 4 min and acute supplementation, an enhancement in performance was observed in six trials and diverse effects in two studies. Due to inconsistent results and methodological aspects of particular studies included in the discussed systematic review (e.g. studied groups – males and females, various sport disciplines, diverse level of training of participants; study protocols − tests evaluating performance, doses of SB, period of supplementation etc.) no clear conclusion regarding duration of exercise and effectiveness of SB supplementation were formulated. Taking into account the results of our study, in team sports disciplines, it is rather the duration of the SB supplementation that determines the ergogenic effect of sodium bicarbonate to a greater extent than duration of effort. In our study the participants from acute- and chronic-supplementation groups were subjected to the same type of efforts and with the same sequence of efforts (WAnT_1 – HST – WAnT_2), yet the results of the efforts differed between groups. Although both groups reached enhancement in HST performance, the solely chronic supplementation group improved in WAnT_1 after SB supplementation.

It could also be worth noting that our study has some specific limitations and strengths. Firstly, the relatively small sample size could be seen as a disadvantage, particularly as the sample demographic is very specific and therefore may not be applicable or comparable to other team sport disciplines. The second limitation associated with this study is the uncertainty of athletes fully adhering to the supplement dose recommendations (particularly the chronic dose). However, we tried to minimize this issue through close cooperation with the coaches of the studied athletes. Moreover, we would also like to underline that potential slight differences in training length between groups should be considered as practically and clinically negligible due to the fact that (i) the total training durations varied between groups by only about 24 min (~ 7.8 vs. ~ 8.2 h) per 8 days, and it would be difficult to expect that this would affect the adaptation of trained athletes in such a short time; (ii) the training load and exercise specifics (Table S1) were performed in a similar manner, due to the fact that recorded training duration differences resulted from the physically inactive time devoted to coaches’ explanations and demonstration of technical and tactical exercise tasks. Therefore, this aspect led to a slight extension of the training duration, however it did not affect the intensity and nature of the effort. Conversely, the strengths of the study are that only trained field hockey athletes were examined in two homogeneous groups. Moreover, blood acid-base balance markers, anaerobic capacity parameters and discipline-specific performance results were utilized, providing highly accurate and reliable data.

## Conclusions

This randomized controlled trial study indicates that sodium bicarbonate supplementation supports discipline-specific performance among field hockey athletes, regardless of whether acute or chronic supplementation doses are utilized. The progressive-chronic protocol appears to have an impact on anaerobic power − especially at the beginning of the exercise and before the occurrence of exercise-induced fatigue. However, the acute protocol significantly affects the buffering capacity, which may determine the athlete’s effectiveness during high-intensity events such as competitions. Moreover, the potential factor of duration on exercise-induced fatigue suggests that subsequent studies investigating sodium bicarbonate supplementation among team sport athletes should concentrate on ascertaining the efficiency of chronic and acute supplementation in varying time frames, using larger sample sizes and among different forms of team sports. Finally, the use and choice of the sodium bicarbonate supplementation protocol, should be ultimately determined by a qualified specialist. These approaches may increase the effectiveness of sodium bicarbonate ergogenic support and at the same time protect against the occurrence of any side effects or health risks, as well as economic losses associated with the purchase of a preparation that, supplemented in the wrong way, could be ineffective.

## Supplementary information

**Additional file 1:Table S1.** Structure of the typical training units specificity of the field hockey players during the study procedures.

**Additional file 2:Table S2.** CONSORT checklist.

## Data Availability

The datasets analyzed for this study can be found in the *Figshare* repository: https://figshare.com/s/5aeb294d2414ce9c5b90 (doi: 10.6084/m9.figshare.11854623).

## Abbreviations

AP_CT_: Average power over the P_CT_
BE: Base excess
bpm: beats per minute
BW: Body weight
GI: Gastrointestinal
HCO_3_¯: Bicarbonate
HST: Specific hockey field test
HR: Heart rate
IST: Intermittent-Sprint Test
La: Lactate
MP: Mean power
PLA: Placebo
P_CT_: Power carry threshold at 97%_PP_
PP: Peak power
SB: Sodium bicarbonate
W: Watts
WAnT: The Wingate anaerobic test
W_EF_: Work effort
