# Supplementary material for: The influence of progressive-chronic and acute sodium bicarbonate supplementation on anaerobic power and specific performance in team sports: a randomized, double-blind, placebo-controlled crossover study
Source: Nutr Metab (Lond). 2020 May 24;17:38. doi: 10.1186/s12986-020-00457-9 (PMC7245907; doi:10.1186/s12986-020-00457-9)
Supplement: Supplementary file 1 — Additional file 1:Table S1. Structure of the typical training units specificity of the field hockey players during the study procedures. [file 12986_2020_457_MOESM1_ESM.docx]

Supplementary Table S1. Structure of the typical training units’ specificity of the field hockey players during the study procedures

| No. | Training section | Range (min) | | |
| --- | --- | --- | --- | --- |
|  |  | Min | − | Max |
| 1. | Warm-up | 15 | − | 20 |
| 2. | Short organizational break and coaches’ explanations of exercise tasks | 2 | − | 5 |
| 3. | Small-sides game | 8 | | |
| 4. | Short organizational break and coaches’ explanations of exercise tasks | 3 | − | 5 |
| 5. | Technical/tactical skills 4/5 exercise tasks* | 6 | − | 8 |
| 6. | Short organizational break and coaches’ explanations of exercise tasks | 3 | − | 5 |
| 7. | Game* | 15 | − | 20 |
| 8. | Post-exercise cool-down phase | 5 | − | 10 |

* In the marked sections of active efforts there were short technical breaks of 2−4 minutes.
